# Anti-TNFα as an Adjunctive Therapy in Pancreas and Kidney Transplantation

**DOI:** 10.3389/ti.2025.14026

**Published:** 2025-03-18

**Authors:** Christophe Masset, Benoit Mesnard, Olivia Rousseau, Alexandre Walencik, Ismaël Chelghaf, Magali Giral, Aurélie Houzet, Gilles Blancho, Jacques Dantal, Julien Branchereau, Claire Garandeau, Diego Cantarovich

**Affiliations:** ^1^ Institut de Transplantation-Urologie-Néphrologie (ITUN), Nantes University Hospital, Nantes, France; ^2^ Nantes Université, INSERM, Center for Research in Transplantation and Translational Immunology, UMR 1064, Nantes, France; ^3^ Laboratoire Human Leucocyte Antigen (HLA), Etablissement Français du Sang, Nantes, France

**Keywords:** anti-TNFα, pancreas transplantation, allograft thrombosis, allograft rejection, ischemia/reperfusion, inflammation

## Abstract

The rate of early pancreas allograft failure remains high due to thrombosis but also to severity of rejection episodes. We investigated if adjunct anti-TNFα therapy was safe and could improve outcomes after pancreas transplantation. We investigated all pancreas transplants performed in our institution between 2010 and 2022. Etanercept, an anti TNFα therapy, was added to our standard immunosuppressive regimen since 2017 after approval from our institutional human ethics committee. Pancreas survival, rejection episodes, as well as infectious complications were analyzed. A total of 236 pancreas transplants were included, among whom 87 received Etanercept for induction. In multivariable analysis, after adjustment on confounding variables, pancreas survival did not differ between groups (HR = 0.92, CI 95% = 0.48; 1.73, p = 0.79). However, patients receiving Etanercept presented a significantly lower occurrence of pancreas rejection in multivariate analysis (HR = 0.36, CI 95% = 0.14; 0.95, p = 0.04). Patients receiving Etanercept did not experienced a higher risk of bacterial, fungal, CMV nor BK virus infections compared to the non-treated group. The use of anti-TNFα after pancreas transplantation was safe and did not increase infectious complications. Despite a similar rate of thrombosis, anti-TNFα significantly reduced pancreatic rejection, thus supporting its use among pancreas transplant recipients.

## Graphical Abstract

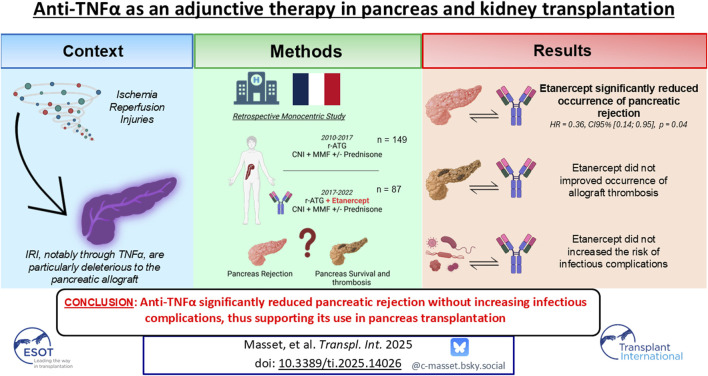

## Introduction

Despite improvement in recent decades, pancreas allografts still face early failure, with approximately 7%–10% experiencing complete thrombosis, leading to significant morbidity and mortality [1–3]. While traditionally categorized as a “technical failure,” its association with prolonged cold ischemia time, along with established risk factors such as donor age and BMI, suggests a connection with an immune response related to ischemia/reperfusion [4–6]. Our group recently described the mechanisms of sterile inflammation further conducing to pancreatic thrombosis and/or rejection [7]. This includes activation of endothelial cells, innate immune cells (neutrophils, monocytes), and platelets [8, 9]. Inflammatory cytokines play a pivotal role in driving the pathophysiological pathways leading to immunothrombosis. Specifically, TNFα acts as a potent activator of endothelial cells and neutrophils, promoting the expression of adhesion molecules, secretion of cytotoxic molecules, and activation of coagulation [10, 11]. In addition, TNFα is well known to promote infiltration of immune cells into allografts and thus promote further rejection [12]. In particular, pancreas allografts are recognized as being very sensitive to alloimmune responses with a high rate of pancreatic loss following a rejection episode [13–15].

Etanercept is a recombinant fusion protein with anti-TNFα activity. It has been used widely as an anti-inflammatory drug for numerous arthritic conditions and used since several years following islet transplantation due to the *in-vitro* toxicity of TNFα on β-cells [16]. Initial reports demonstrated promising results, including high rates of insulin independence at 1 year [17]. Consistent with these findings, Etanercept is currently extensively used among islet transplant centers, as it may facilitate islet engraftment by mitigating the innate inflammatory response observed during ischemia/reperfusion but also reduce occurrence of rejection [18].

Drawing from the experience of islet transplant recipients, we opted several years ago to modify the immunosuppressive strategy in pancreas transplant recipients by incorporating Etanercept during the early post-operative period. Indeed, blocking TNFα in the early post-transplantation period appears to be a very promising strategy, as it helps reduce the cytokine storm associated with ischemia-reperfusion injury and the subsequent risk of allograft rejection. This approach is particularly relevant in the context of pancreatic transplantation, given the highly inflammatory nature of the digestive segment transplanted alongside the pancreas to ensure exocrine drainage. We thus hypothesized that an anti-TNFα therapy may be beneficial by reducing activation of immune system following ischemia/reperfusion, and thus reduce occurrence of pancreas rejection and immunological thrombosis.

Here, we present an evaluation of the outcomes of anti-TNFα therapy as an adjunctive treatment to prevent rejection in a large single-center cohort of pancreas transplant recipients.

## Materials and Methods

### Studied Population

All patients who underwent pancreas transplantation (simultaneous pancreas-kidney (SPK), pancreas after kidney (PAK), and pancreas transplant alone (PTA) between 1st January 2010, and 30th April 2022, at our institution were included in the study. Data were extracted from the French prospective DIVAT cohort of transplanted patients.^1^

### Available Data

Complete available data are presented in Table 1. Donor and recipient characteristics, as well as peri-transplant parameters, were prospectively collected. Pancreas failure was defined by either the persistence of insulin requirement, allograft removal, or retransplantation. Kidney failure was defined by either a return to dialysis or retransplantation. Rejection episodes were diagnosed based on pancreatic biopsy findings or if no biopsy was available, pancreas rejection was considered in cases of dysfunction (hyperglycemia + increase in lipase levels) with a biopsy-proven diagnosis of kidney rejection [19]. This strategy aimed to minimize unnecessary invasive biopsies, especially for the pancreatic allograft. Rejection episodes were categorized according to the Banff classification. Cellular rejection was usually treated with steroid pulses or r-ATG (Thymoglobulin), while humoral rejection was managed with plasma exchanges, intravenous immunoglobulins, and sometimes associated with CD20 depleting therapy. Donor specific antibodies (DSA), assessed pre-transplant, in case of rejection, and at 1 year post-transplantation were determined by Luminex^®^ assay and considered positive when mean fluorescence index values were superior to 1000. Infectious complications, including CMV viremia (either asymptomatic or associated with CMV disease), BK virus (BKV) viremia (either asymptomatic or associated with BKV nephropathy), fungal infections, and severe bacterial infections, were recorded. Prospective follow-up of pancreatic and kidney allograft functions included fasting glycemia, fasting C-peptide, HbA1c levels, estimated glomerular filtration rate (eGFR, using the CKD-EPI formula), collected every 3 months during the first year and then annually. Follow-up and data collection ceased upon transplant failure or death.

**TABLE 1:** Description of the studied cohort depending on the administration of Anti-TNFα in the early post-operative time (p-values are obtained using Chi-square test or Fisher exact test for categorical variables and using Student’s t-test or Mann-Whitney U for continuous variables).

	Whole cohort (n = 236)			Anti-TNFα (n = 87)			Standard of care (n = 149)			p-value
	NA	N	%	NA	n	%	NA	N	%	
Type of graft	0			0			0			
SPK		182	77.1		72	82.7		110	73.8	0.1481
PAK		22	9.3		4	4.6		18	12.1	0.0651
PTA		32	13.6		11	12.6		21	14.1	0.8451
Male recipient	0	133	56.3	0	46	52.9	0	87	58.4	0.4181
Retransplantation	0	29	12.3	0	8	9.2	0	21	14.1	0.3096
Pancreas preservation fluid	13			3			10			
Celsior		65	29.2		8	9.5		57	41.0	<0.0001
IGL		89	39.9		53	63.1		36	25.9	<0.0001
Other		69	30.9		23	27.4		46	33.1	0.4560
Male donor	0	157	66.5	0	56	64.4	0	101	67.8	0.2350
Vascular cause of donor death	0	92	38.9	0	34	39.1	0	58	38.9	>0.9999
Donor hypertension history	0	16	7.2	9	5	6.4	5	11	7.3	0.7572
History of donor cardiac arrest sampling	0	61	25.1	1	25	29.1	1	36	24.3	0.4431
Use of vasopressive drug	0	203	89.4	8	74	93.7	1	129	87.2	0.1741
Depleting induction	0	218	92.4	0	87	100	0	131	87.9	0.0002
Initial maintenance therapy	0			0						
Cyclosporine		2	0.8		0	0	0	2	1.3	0.5325
Tacrolimus		234	99.1		87	100	0	147	98.6	0.5325
Antiproliferative drugs		235	99.6		87	100	0	148	99.3	>0.9999
mTOR inhibitors		0	0		0	0	0	0	0	>0.9999
Oral steroids		231	97.9		87	100	0	144	96.6	0.2963
Pre-formed DSA	0	25	10.6	0	10	11.5	0	15	10.4	0.6587
	NA	Mean	SD	NA	Mean	SD	NA	Mean	SD	
Recipient age (years)	0	40.6	7.9	0	39.6	7.3	0	41.3	8.3	0.1104
Recipient BMI (kg/m^2^)	0	23.7	3.7	0	23.9	3.8	0	23.6	3.6	0.3313
Duration of diabetes (years)	8	26.4	8.7	8	24.6	8.8	0	27.4	8.5	0.0276
Pancreas CIT (min)	0	608	140	0	563	136	1	635	136	<0.0001
Kidney CIT (min)	0	753	155	0	688	133	0	794	154	<0.0001
Duration in ICU at post-op (days)	6	1.7	1.7	6	1.4	0.9	0	1.9	1.9	0.0194
Donor age (years)	0	32.9	10.9	0	33.1	11.2	0	32.7	10.8	0.7978
Donor BMI (kg/m^2^)	0	23.1	3.0	0	22.8	2.9	0	23.2	3.1	0.4103
Donor creatininemia (µmol/L)	0	77	33	0	80	40	0	76	28	0.8970

BMI, body mass index; eGFR, estimated glomerular filtration rate; ICU, intensive care unit; NA, not available (missing); PAK, pancreas after kidney; PTA, pancreas transplant alone; SD, standard deviation; SPK, simultaneous pancreas-kidney; CIT, Cold Ischemia Time.

### Immunosuppressive Protocol

The management of pancreas transplantation was consistent across all categories (SPK, PAK, and PTA) and remained globally unchanged during the study period, except for the addition of Etanercept. The surgical technique remains globally unchanged during the study period, with digestive anastomosis performed in all cases for exocrine diversion. Induction therapy consisted mostly in rabbit antithymocyte globulin (rATG) for five alternate days, or either basiliximab in some rare cases, along with two pulses of 500 mg methylprednisolone. From April 2017, pancreas transplant recipients received an additional course of Etanercept at a similar dosage than for islet recipients: 50 mg on day 0 (intravenous), followed by 25 mg (subcutaneous) on days 3, 7, and 10. All patients underwent screening for latent tuberculosis and hepatitis viruses before Etanercept administration. Maintenance immunosuppressive therapy included a calcineurin inhibitor (mainly tacrolimus) and mycophenolate mofetil or mycophenolic acid, with oral prednisone tapered and withdrawn from postoperative day 7. Our anticoagulation protocol involved per-operative administration of intravenous aspirin (250 mg) and heparin (25 UI/kg) at the time of clamping, followed by preventive anticoagulation using low molecular weight heparin within the first days post-surgery, typically for 10 days. In the absence of allograft thrombosis, detected on purpose or by systematic CT-scan on day 10, preventive heparin was replaced by long-term administration of antiplatelet therapy. Finally, our strategy for treating pancreatic rejection episodes remained largely consistent throughout the study period (i.e., steroid pulses for cellular rejection, with rATG used in cases of grade II or grade III cellular rejection or steroid resistance, and plasma exchange, IV Ig and Rituximab for treatment of humoral rejection).

### Statistical Analyses

The characteristics at transplantation were described using frequency and proportion for categorical variables and mean and standard deviation for continuous variables. To assess the impact of anti-TNFα treatment on a specific phenotype over time, survival curves were generated using the Kaplan-Meier estimator. Statistical comparisons were conducted using the log-rank test. For univariate analysis, the Student’s t-test or Mann-Whitney test was employed, while multivariate analysis used the Cox model. The anti-TNFα variable was consistently included in the statistical models to evaluate its effect on the different studied outcomes. Initial variable selection was performed retaining only those with a p-value of less than 0.2 according to the Wald test for inclusion in the final Cox model [20]. In addition, five variables were forced selectively into the Cox model for pancreas survival due to their known association with complete thrombosis (pancreas cold ischemia time, and donor-related variables: age, BMI, vascular cause of death, and history of hypertension). Similarly, induction therapy (r-ATG or Basiliximab) was forced into the Cox model for pancreatic rejection. Subsequently, a stepwise forward selection process was conducted, whereby variables were added to the model if their inclusion improved the Bayesian information criterion. The final model comprised the forced variables along with any additional selected variables. Of note, patients with missing data on the variables of interest were excluded from the final analysis. The hazard proportionality assumption was tested from the Schoenfeld residuals [21]. The absence of multicollinearity of the model was verified using the Variance Inflation Factor. To visualize the results, adjusted survival curves were generated to observe the impact of anti-TNFα use over time while holding other variables constant. While one-year endpoints were assessed to accurately determine the impact of anti-TNFα, we also conducted a three-year analysis to gain insights into its long-term effects. Even if some confounding factors may arise well after the induction treatment; these are part of the causal pathway of the initial treatment (i.e., they result from it) and should be considered as part of the evaluation process.

The analysis was conducted using R version 4.1.3, with statistical significance defined as a p-value of less than 0.05.

### Ethical Consent

All data were extracted from the Nantes DIVAT database. This study received data privacy approval from CNIL (09-17-2004, number n°891735, Réseau DIVAT:10.16.618). The patient’s non-opposition regarding access to their medical records, collection and data processing is mandatory under French law. All data were anonymized before analysis. The use of Etanercept in pancreas transplant recipients was approved by the local human ethics committee (n°23-115-09-211). The quality of the DIVAT data bank is validated by an annual audit. The clinical and research activities being reported are consistent with the Principles of the Declaration of Istanbul as outlined in the Declaration of Istanbul on Organ Trafficking and Transplant Tourism.

## Results

### Description of the Population

During the study period, 236 pancreas transplant recipients were included, among whom 87 received anti-TNFα and 149 received standard of care (SOC). The complete characteristics of the population are described in Table 1. Briefly, 77.1% received simultaneous pancreas-kidney (SPK) transplants, 13.6% received pancreas transplant alone (PTA), and 9.3% received pancreas after kidney (PAK) transplants, with a mean age of 40 years. The mean donor’s age was 33 years, with a mean BMI of 23, and 39% of them died from cardiovascular events, without any significant differences observed among groups. Of note, patients receiving anti-TNFα were more often transplanted with shorter pancreatic and kidney cold ischemia times (563 vs. 635 min, p < 0.0001 and 688 vs. 794 min, p < 0.0001 respectively). 10.6% of patients presented with preformed donor-specific antibodies (DSA) at the time of transplantation. Induction therapy consisted of a T-cell depleting agent in 92.4% of the cohort, followed by maintenance therapy comprising a calcineurin inhibitor (mainly tacrolimus: 99.1%) and an antiproliferative agent, either mycophenolate mofetil or mycophenolic acid (99.6%). Oral steroids were administered to 97.9% of patients, followed by rapid tapering during the first weeks post-transplantation.

### Impact of Anti-TNFα on Allograft Survival and Function

**FIGURE 1:**
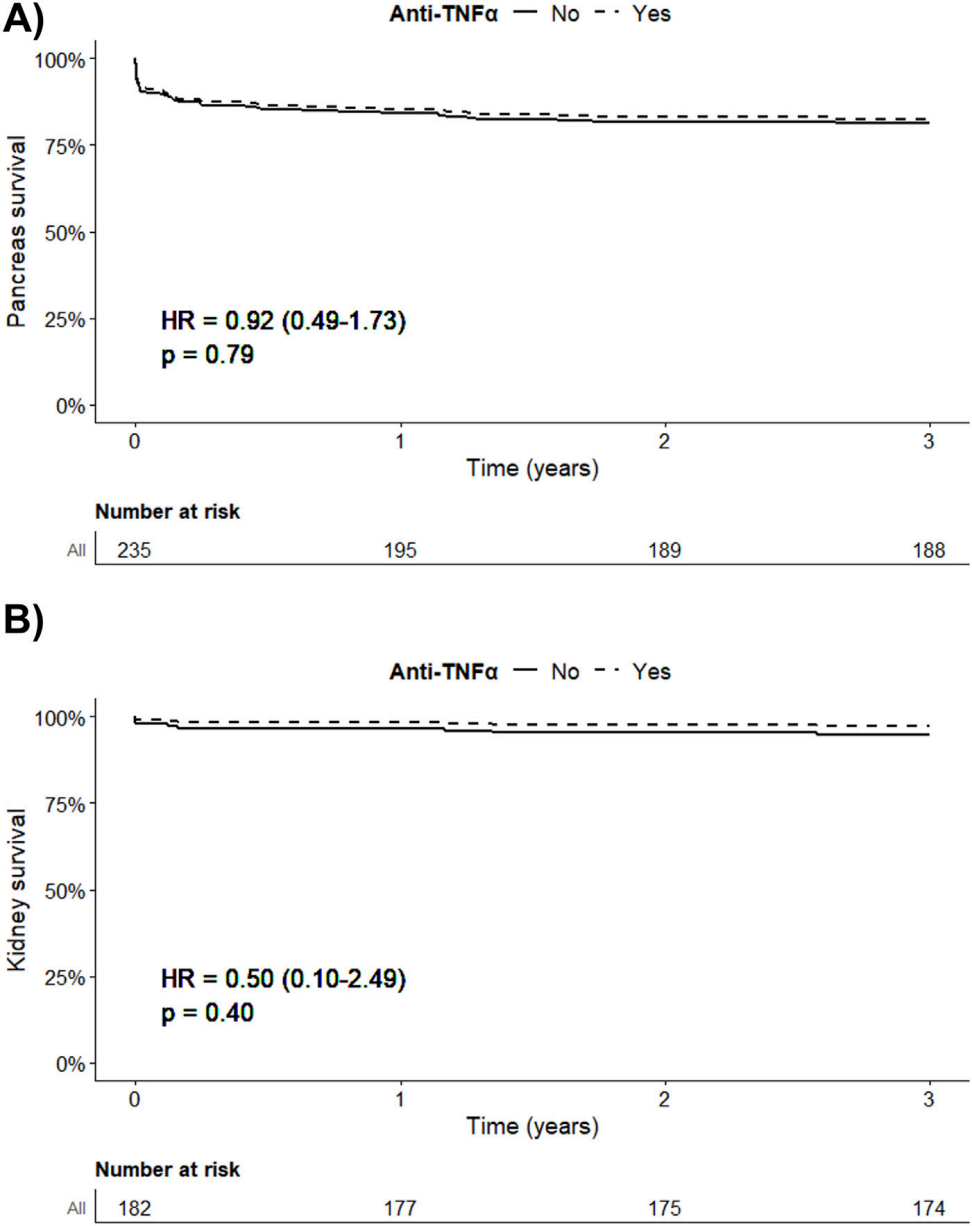
**(A)** Confounder-adjusted death-censored pancreas allograft survival according to the administration of anti-TNFα. **(B)** Confounder-adjusted death-censored kidney allograft survival according to the administration of anti-TNFα among the SPK recipients.

At 3 years post-transplantation, the overall pancreatic allograft survival rate was 80.1%. The main causes of failure were allograft thrombosis (68.1%), allograft rejection (17.0%), and surgical complications (10.6%). Numerically, there were 15 allograft failures in the anti-TNFα group (17.2%, of whom 13/15 were complete thrombosis) and 32 in the SOC group (21.5%, of whom 19/32 were complete thrombosis) at 3 years. After adjusting for confounding variables and factors associated with allograft failure due to thrombosis, the adjusted hazard ratio (HR) for pancreas survival was 0.92 (95% CI = 0.49; 1.73, p = 0.79) for patients receiving anti-TNFα therapy, Table 2. The cumulative adjusted probability of pancreatic allograft survival is presented in Figure 1A. Among SPK recipients, the adjusted HR for kidney allograft survival was 0.50 (95% CI = 0.10; 2.49, p = 0.40) for patients receiving anti-TNFα therapy compared to the SOC group*,* Supplementary Table S1. The cumulative adjusted probability of kidney allograft survival is presented in Figure 1B.

**TABLE 2:** Univariate and multivariate cause-specific Cox model associated with the risk of pancreas graft failure at 3 years post-transplantation. The following variables were forced into the multivariate model due to their known association with pancreas failure: pancreas cold ischemia time, donor age, donor BMI, donor vascular cause of death, donor history of hypertension (47 events were observed during follow-up, 1 observation was excluded because of missing data).

	Univariate analysis			Multivariate analysis		
	HR	95% CI	p-value	HR	95% CI	p-value
Anti-TNFα	0.80	0.43–1.48	0.480	0.92	0.49–1.73	0.7880
Pancreas Cold Ischemia Time	1.00	1.00–1.00	0.016	1.002	1.001–1.004	0.0335
Donor’s age	1.00	0.98–1.03	0.771	1.01	0.98–1.04	0.4479
Donor’s BMI	1.00	0.91–1.10	0.991	1.00	0.91–1.11	0.8978
Donor’s vascular cause of death	0.88	0.48–1.59	0.663	0.54	0.25–1.17	0.1190
Donor’s history of hypertension	1.27	0.45–3.53	0.652	1.43	0.47–4.35	0.5251
Donor’s gender (Female)	1.81	1.02–3.21	0.043	1.90	1.02–3.53	0.0424
Type of transplant: SPK	0.56	0.31–1.02	0.058			
T cell depleting induction	1.91	0.46–7.88	0.370			
Recipient’s age	1.00	0.97–1.04	0.870			
Recipient’s gender (Female)	1.64	0.92–2.91	0.092			
Recipient’s BMI	1.06	0.98–1.14	0.128			
Preemptive SPK	1.12	0.77–1.63	0.541			
Retransplantation	1.59	0.74–3.39	0.235			
Duration of diabetes	1.01	0.97–1.04	0.742			
Pretransplant C peptide	0.95	0.73–1.23	0.678			
Pretransplant HbA1c	0.98	0.80–1.19	0.822			
Donor’s cardiac arrest	0.63	0.31–1.31	0.218			
Donor’s eGFR	1.01	1.00–1.02	0.217			
Use of vasopressive drugs	0.88	0.35–2.23	0.782			
Number of HLA mismatches	1.16	0.86–1.57	0.325			
Use of Cyclosporine (Ref: Tacro)	2.31	0.72–7.42	0.162			
Use of non CNI treatment	0.56	0.08–4.06	0.566			
Anti HLA class I at baseline	1.34	0.70–2.56	0.375			
Anti HLA class II at baseline	0.76	0.35–1.64	0.479			
DSA at baseline	1.17	0.49–2.78	0.718			

We further investigated pancreatic and kidney allograft function censored for allograft loss (Supplementary Figure S1). Regarding the pancreas, no differences were found in fasting glycemia, fasting C-peptide levels, and HbA1c levels during the first 3 years post-transplantation in the anti-TNFα group vs. SOC. Similarly, in the subgroup of SPK recipients, eGFR were globally comparable even if we observed a higher eGFR slope between 3 months and 3 years among patients from the SOC group vs. anti-TNFα (respectively −12.1% and −2.3%).

### Impact of Anti-TNFα on Occurrence of Rejection and *De Novo* DSA

**FIGURE 2:**
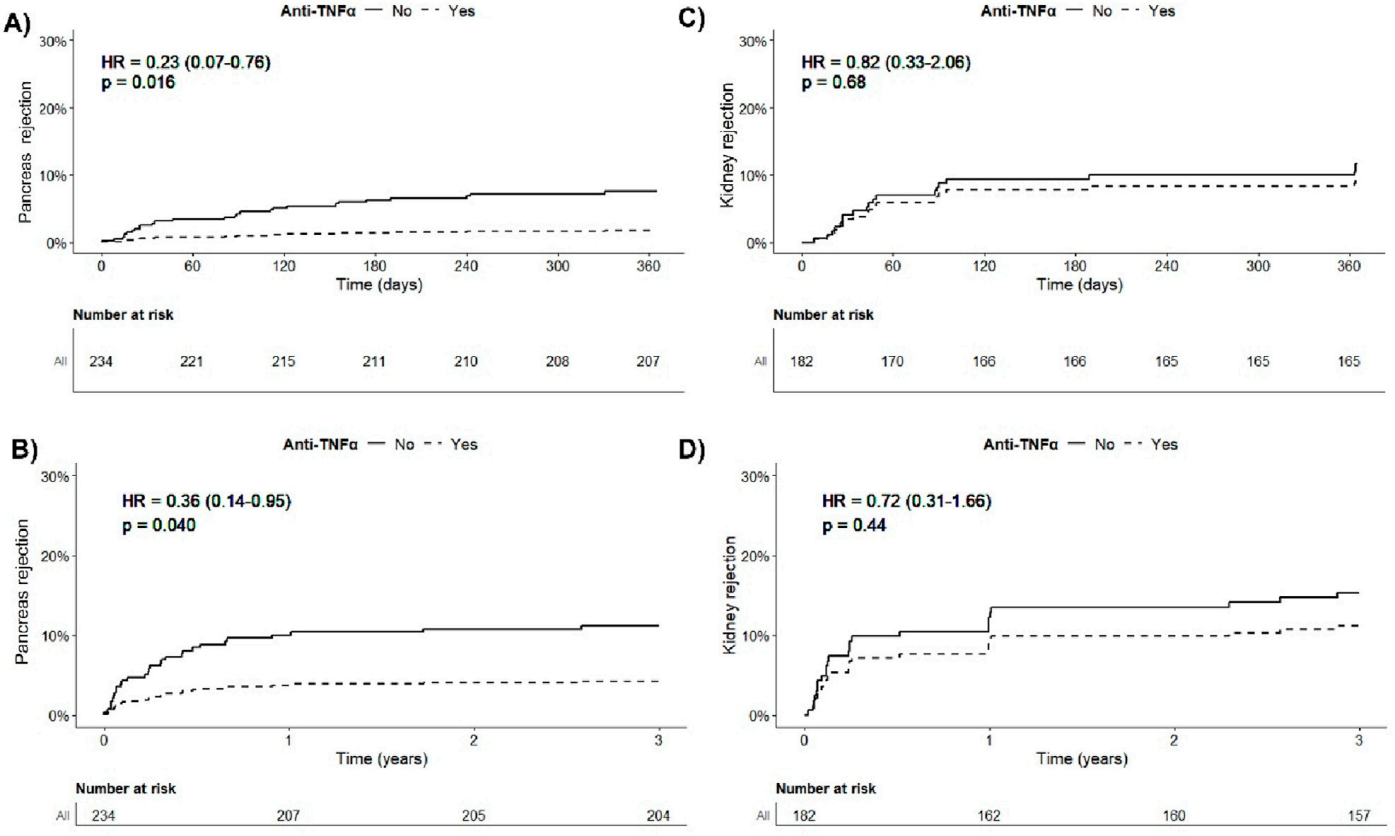
**(A)** Confounder-adjusted death-censored occurrence of pancreas rejection according to the administration of anti-TNFα during the first year post-transplantation. **(B)** Confounder-adjusted death-censored occurrence of pancreas rejection according to the administration of anti-TNFα at 3 years post-transplantation. **(C)** Confounder-adjusted death-censored occurrence of kidney rejection according to the administration of anti-TNFα among SPK recipients during the first year post-transplantation. **(D)** Confounder-adjusted death-censored occurrence of kidney rejection according to the administration of anti-TNFα among SPK recipients at 3 years post-transplantation.

**FIGURE 3:**
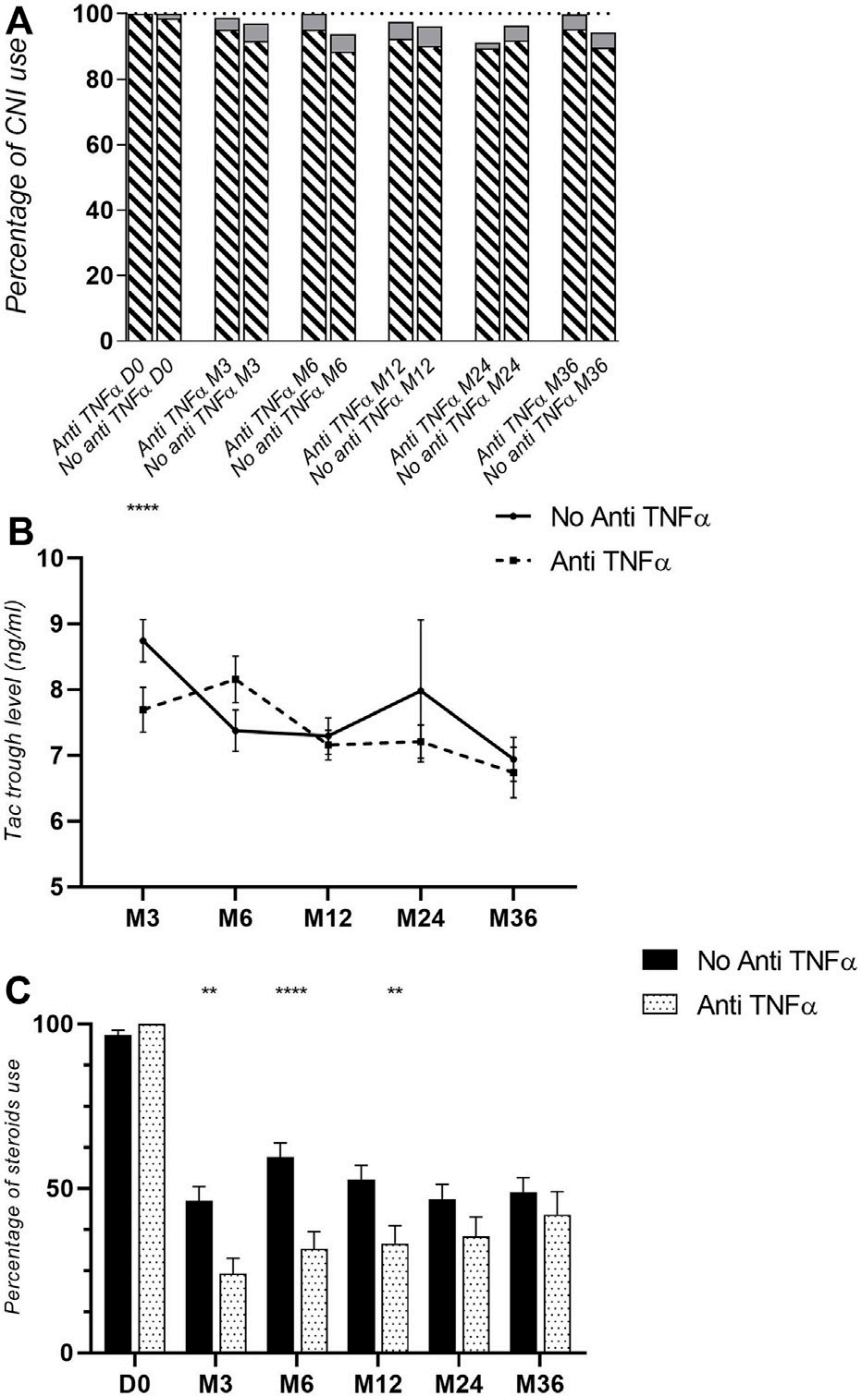
Representation of maintenance therapy during the first year post-transplantation. **(A)** Percentage of calcineurin inhibitors use (tacrolimus: crosshatched bars and cyclosporine: Gy bars). **(B)** Tacrolimus trough levels. **(C)** Use of steroids. *: p < 0.05; **: p < 0.01, ***: p < 0.001, ****: p < 0.0001.

At 3 year post-transplantation, there were 5 pancreatic rejection episodes (5.7%) diagnosed in the anti-TNFα group (3 proven by pancreatic biopsy) and 26 (17.4%) in the SOC group (17 proven by pancreatic biopsy). The complete description of these rejection episodes is provided in Table 3. The occurrence of a pancreatic rejection episode led to further allograft loss in around 60% of cases. In the multivariate analysis, after adjusting for confounding factors—particularly induction therapy—adjunctive treatment with anti-TNFα was significantly protective against the occurrence of pancreatic rejection during the first year post-transplantation (HR = 0.23, 95% CI = 0.07–0.76, p = 0.01; Table 4; Figure 2A). Importantly, this protective effect persisted over time and remained significant up to 3 years post-transplantation (HR = 0.36, 95% CI = 0.14–0.95, p = 0.04; Table 5; Figure 2B). Notably, among the 18 patients who received non-depleting induction therapy and no anti-TNFα, the incidence of pancreatic rejection at 3 years was 11.1%, which aligns with the rejection incidence in patients who received a T-cell depleting induction without anti-TNFα. This may be linked to a higher level of maintenance immunosuppressive burden administered during the first year in these patients (Supplementary Figure S2). Finally, occurrence of DSA at 1 year was comparable between groups (16.4% vs. 10.4%, p = 0.55). The protective effect of anti-TNFα on pancreatic rejection was particularly notable as maintenance therapy was significantly reduced in the anti-TNFα group compared to the SOC group, especially regarding tacrolimus trough levels and steroid use during the first months, Figure 3.

**TABLE 3:** Description of pancreatic rejection episodes occurring in the studied period and their long-term evolution, depending on the administration or not of anti-TNFα.

	Anti-TNFα (n = 5)			No anti-TNFα (n = 26)		
	NA	N	%	NA	n	%
TCMR	0	1	20	0	10	38.5
Allograft loss post-TCMR	0	0	0	0	5	50
ABMR	0	2	40	0	7	27
Allograft loss post-ABMR	0	1	50	0	4	57
Mixed rejection	0	2	40	0	9	34.5
Allograft loss post Mixed rejection	0	2	100	0	5	55
All pancreatic loss post-rejection	0	3	60	0	14	54

**TABLE 4:** Univariate and multivariate cause-specific Cox model associated with the risk of pancreas graft rejection in the first year post-transplantation. The type of induction therapy variable was forced into the multivariate model due to its known association with pancreas rejection (27 events were observed during follow-up, 0 observations were excluded because of missing data).

	Univariate analysis			Multivariate analysis		
	HR	95% CI	p-value	HR	95% CI	p-value
Anti-TNFα	0.20	0.06–0.66	0.008	0.23	0.07–0.75	0.0161
Type of transplant: SPK	0.24	0.11–0.52	0.001	0.29	0.13–0.62	0.0015
T cell depleting induction	1.02	0.24–4.29	0.983	0.96	0.22–4.21	0.9569
Donor’s gender (Female)	2.28	1.07–4.86	0.032	2.31	1.08–4.95	0.0305
Recipient’s gender (Female)	1.20	0.57–2.56	0.631			
Recipient’s age	1.00	0.96–1.05	0.930			
Recipient’s BMI	1.05	0.95–1.15	0.335			
Preemptive SPK	0.54	0.29–0.99	0.047			
Pancreas Cold Ischemia Time	1.00	1.00–1.01	0.030			
Retransplantation	2.19	0.88–5.42	0.091			
Duration of diabetes	1.00	0.96–1.05	0.846			
Pretransplant C peptide	0.58	0.24–1.39	0.222			
Pretransplant HbA1c	1.28	1.06–1.55	0.010			
Donor’s age	1.04	1.00–1.07	0.043			
Donor’s BMI	1.12	0.99–1.27	0.077			
Donor’s vascular cause of death	1.29	0.60–2.75	0.516			
Donor’s history of hypertension	0.99	0.23–4.18	0.989			
Donor’s cardiac arrest	0.22	0.05–0.91	0.037			
Donor’s eGFR	1.00	0.98–1.01	0.551			
Use of vasopressive drugs	1.46	0.35–6.19	0.606			
Number of HLA mismatches	1.20	0.80–1.78	0.376			
Use of Cyclosporine (Ref: Tacro)	7.37	2.54–21.35	0.001			
Use of non CNI treatment	4.34	1.30–14.41	0.017			
Anti HLA class I at baseline	1.72	0.77–3.85	0.190			
Anti HLA class II at baseline	0.86	0.32–2.30	0.768			
DSA at baseline	0.97	0.29–3.23	0.954			

**TABLE 5:** Univariate and multivariate cause-specific Cox model associated with the risk of pancreas graft rejection in the first 3 years post-transplantation. The type of induction therapy variable was forced into the multivariate model due to its known association with pancreas rejection (30 events were observed during follow-up, 2 observations were excluded because of missing data).

	Univariate analysis			Multivariate analysis		
	HR	95% CI	p-value	HR	95% CI	p-value
Anti-TNFα	0.32	0.12–0.83	0.019	0.36	0.14–0.95	0.0396
Type of transplant: SPK	0.26	0.13–0.53	0.001	0.29	0.14–0.59	0.0008
T cell depleting induction	1.14	0.27–4.79	0.856	1.15	0.27–4.99	0.8484
Recipient’s age	1.00	0.95–1.04	0.9			
Recipient’s gender (Female)	1.3	0.64–2.66	0.474			
Recipient’s BMI	1.02	0.93–1.12	0.612			
Preemptive SPK	0.67	0.39–1.14	0.14			
Pancreas Cold Ischemia Time	1.00	1.00–1.01	0.025			
Retransplantation	2.35	1.01–5.48	0.048			
Duration of diabetes	1.01	0.96–1.05	0.785			
Pretransplant C peptide	0.59	0.27–1.31	0.197			
Pretransplant HbA1c	1.25	1.03–1.50	0.022			
Donor’s age	1.03	1.00–1.07	0.045			
Donor’s gender (Female)	2.13	1.04–4.35	0.039			
Donor’s BMI	1.09	0.97–1.23	0.155			
Donor’s vascular cause of death	1.07	0.52–2.22	0.854			
Donor’s history of hypertension	0.89	0.21–3.72	0.870			
Donor’s cardiac arrest	0.54	0.21–1.40	0.204			
Donor’s eGFR	1.00	0.98–1.01	0.691			
Use of vasopressive drugs	1.65	0.39–6.93	0.495			
Number of HLA mismatches	1.18	0.81–1.72	0.392			
Use of Cyclosporine (Ref: Tacro)	6.78	2.36–19.49	0.001			
Use of non CNI treatment	3.91	1.18–12.89	0.025			
Anti HLA class I at baseline	1.46	0.67–3.22	0.342			
Anti HLA class II at baseline	0.94	0.38–2.32	0.892			
DSA at baseline	0.85	0.26–2.80	0.784			

Conversely, anti-TNFα did not significantly impact the risk of kidney rejection (HR = 0.72, 95% CI = 0.31; 1.66, p = 0.44), as shown in Figures 2C, D and Supplementary Tables S2, S3 *.* Nevertheless, we observed a shift in the kidney Banff classification, with a trend toward fewer TCMR and ABMR and more Borderline lesions among SPK patients treated with anti TNFα, Supplementary Figure S3 *.*

### Impact of Anti-TNFα on Occurrence of Infectious Complications

**FIGURE 4:**
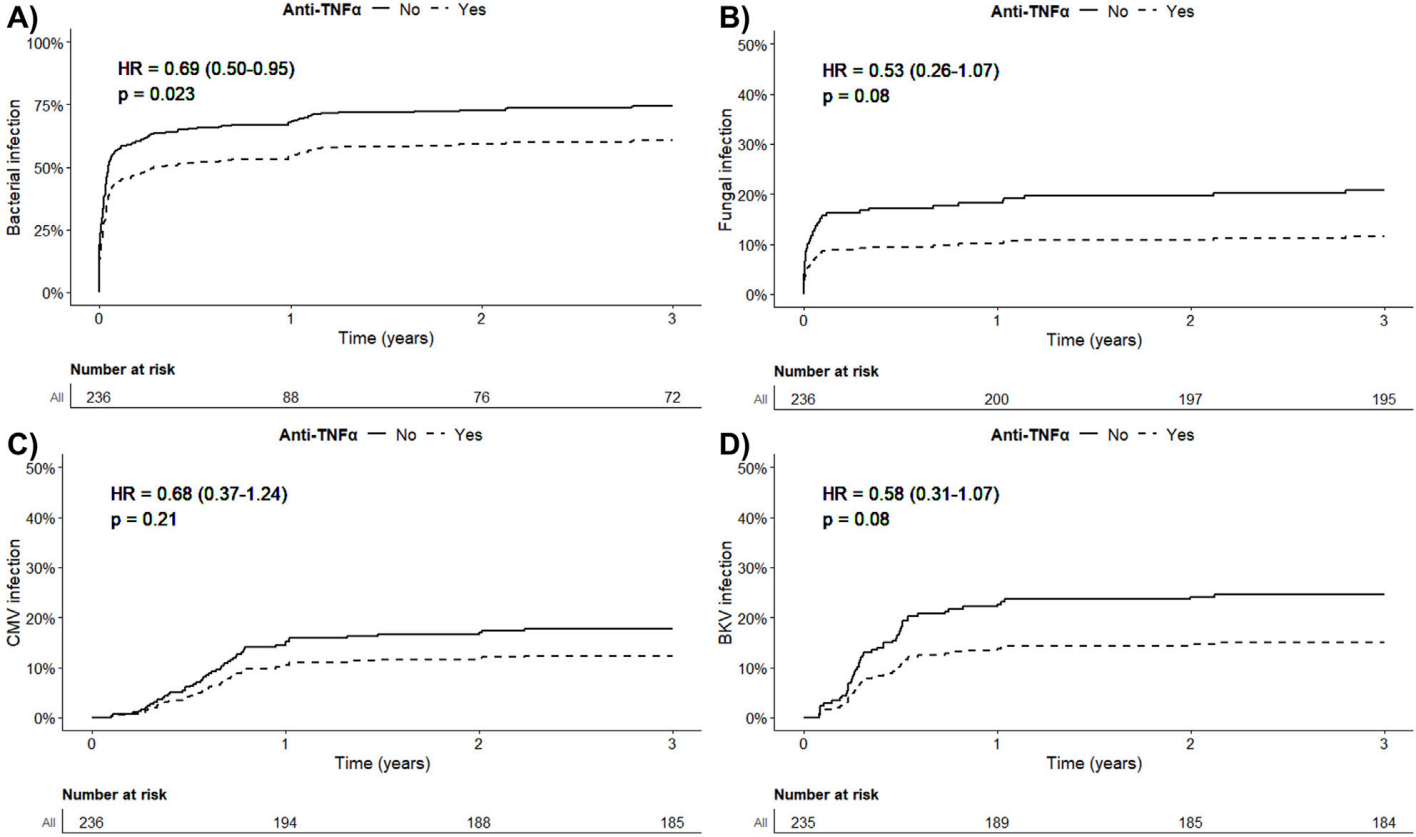
**(A)** Confounder-adjusted death-censored occurrence of a severe bacterial infection during the first year post-transplantation according to the administration of anti-TNFα. **(B)** Confounder-adjusted death-censored occurrence of a fungal infection during the first year post-transplantation according to the administration of anti-TNFα. **(C)** Confounder-adjusted death-censored occurrence of a CMV viremia during the first year post-transplantation according to the administration of anti-TNFα. **(D)** Confounder-adjusted death-censored occurrence of a BK virus viremia during the first year post-transplantation according to the administration of anti-TNFα.

During the first year post-transplantation, we did not observe an increased risk of infectious complications following the administration of anti-TNFα. Regarding the occurrence of severe bacterial infections, the adjusted HR was 0.69, 95% CI = 0.50; 0.95, p = 0.02 for patients receiving anti-TNFα, as shown in Figure 4A, and Supplementary Tables S4, S5 *.* Concerning the occurrence of fungal infections, the adjusted HR was 0.53, 95% CI = 0.26; 1.07, p = 0.08 for patients receiving anti-TNFα, as depicted in Figure 4B and Supplementary Tables S6, S7 *.* The risk of CMV viremia was similar among patients receiving anti-TNFα compared to others (adjusted HR = 0.89, 95% CI = 0.37; 1.24, p = 0.21), Figure 4C and Supplementary Tables S8, S9 *.* Finally, the risk of BKV viremia was also similar following the administration of anti-TNFα (HR = 0.58, 95% CI = 0.31; 1.07, p = 0.08), Figure 4D, Supplementary Tables S10, S11. No cases of tuberculosis or viral hepatitis replication were observed among patients having received anti-TNFα therapy. Finally, anti-TNFα therapy did not impact patient survival (Supplementary Figure S4).

## Discussion

Our study highlights for the first time the significant reduction in the incidence of pancreatic rejection among patients who received anti-TNFα during the first week following pancreas transplantation. This result is all the more notable given that the maintenance therapy in the anti-TNFα group was significantly less intense, particularly with regard to tacrolimus trough levels and the use of oral steroids. Other published *in-vitro* data have reported the benefit of early treatment using anti-TNFα for reducing cytokine storm and leukocyte infiltration in the allograft [11, 12, 22, 23]. However, to the best of our knowledge, no clinical data in humans support its use for the prevention of rejection. This result is all the more important as the occurrence of pancreas rejection exacerbates further allograft loss [24–26], which was not attenuated by anti-TNFα therapy in our series. The effect of anti-TNFα therapy on pancreas rejection might be linked to the duodenal part of the pancreatic allograft which might trigger important inflammatory reactions and further alloimmune responses [27]. The benefit of TNFα blockade for digestive inflammatory diseases has been well known for several years [28, 29]. Anti-TNFα therapy has also been used in some cases of refractory intestinal rejection episodes to allow resolution of the alloimmune response [30]. In recipients of a pancreas transplant, a correlation between duodenal rejection and pancreatic rejection has been observed in some cases, suggesting possible interconnected mechanisms [31–33]. This hypothesis is moreover supported by the absence of a significant effect of anti-TNFα on the incidence of kidney allograft rejection. Finally, the observed trend toward a higher incidence of humoral/mixed rejection in patients who received anti-TNFα warrants further investigation and close monitoring to assess the potential for more severe rejection episodes in these patients. In the context of pancreatic transplantation, basic science data regarding the specific effects of anti-TNFα blockade will be of great interest.

Nevertheless, despite the addition of anti-TNFα, we did not observe an improvement in pancreatic allograft survival nor thrombosis. This is certainly due to the complex pathophysiology of pancreatic allograft thrombosis, which involve both immune and non-immune mechanisms [6, 34, 35], as well as implication of multiple inflammatory cytokins such as IL1β. In islet transplantation, the combination of anti-TNFα and anti-IL-1β has proven to be effective in improving grafted islets and long-term survival [36, 37], whereas the use of Etanercept alone did not benefit islet survival [38]. This is consistent with murine models, which report a synergy in the blockade of anti-TNFα and IL-1β regarding islet survival, whereas their respective effects were low independently [39]. Further research on the pathophysiology of pancreas thrombosis will undoubtedly allow a better understanding of this complication and an improvement in strategies to prevent its occurrence.

Importantly, we observed an overall safety profile of anti-TNFα in pancreas transplant recipients. Notably, we did not observe any increase in the risk of severe bacterial or fungal infections, CMV viremia, nor BKV viremia. We even observed a trend towards fewer infectious complications, which can be explained by a reduced maintenance immunosuppressive treatment in patients receiving anti-TNFα. This contrasts with previously reported data in kidney transplant recipients [40, 41] but aligns with findings in liver transplantation [42]. Differences in maintenance therapy, particularly the use of steroids, might explain these discrepancies. Furthermore, although anti-TNFα has been reported to induce rare cases of renal injuries [43], our patients did not exhibit worsened kidney allograft function.

Our study has several limitations, the most significant being its retrospective, single-center design, which may introduce unforeseen confounding factors due to variations across different time periods. However, it is important to note that during the study period, there were no major changes in our surgical techniques or perioperative management of pancreas transplant recipients, except for the use of anti-TNFα and the administration of basiliximab as induction therapy in a small proportion of non-immunized patients. The differences in the initial use of a T-cell-depleting agent, stemming from a local protocol implemented in our center in 2014 to reserve Thymoglobulin for the treatment of pancreatic acute rejection episodes, may have introduced a potential confounding bias regarding rejection occurrence. However, we observed a similar incidence of rejection among patients who did not receive a T-cell-depleting agent compared to those who did. Furthermore, the use of T-cell-depleting agents was accounted for and adjusted in our multivariate analysis, ensuring that the observed difference in rejection rates is attributable to anti-TNFα rather than variations in the use of T-cell-depleting agents.

Additionally, the lack of systematic pancreatic biopsies, either for cause or protocolar, may introduce bias in the definition of rejection episodes. Nevertheless, in our cohort, the rate of biopsy-proven pancreatic rejection compared to the global rate of diagnosed rejection was similar among patients receiving anti-TNFα compared to others, suggesting a relatively low impact on our final results.

Finally, it will be of great interest to confirm the benefit of anti-TNFα therapy in pancreas transplant recipients in a multicenter prospective study.

In conclusion, we report the first use of anti-TNFα adjunctive therapy in pancreas transplantation. Although it did not improve neither the rate of early failure due to thrombosis nor overall allograft survival, anti-TNFα significantly reduced the occurrence of pancreatic rejection without increasing infectious complications. Given the retrospective monocentric of our cohort, further evaluation of anti-TNFα would be of interest to properly define its role in pancreas transplantation.

## Data Availability

The data analyzed in this study is subject to the following licenses/restrictions: Data are available upon reasonable request to the corresponding author. Requests to access these datasets should be directed to christophe.masset@univ-nantes.fr.

## Footnotes

1
http://www.divat.fr

## Abbreviations

SPK, Simultaneous Pancreas-Kidney; PAK, Pancreas After Kidney; PTA, Pancreas Transplantation Alone; DSA, Donor Specific Antibodies; CIT, Cold Ischemia Time; eGFR, estimated Glomerular Filtration Rate; SOC, Standard of Care; HR, Hazard Ratio.
