# Supplementary material for: Anti-TNFα as an Adjunctive Therapy in Pancreas and Kidney Transplantation
Source: Transpl Int. 2025 Mar 18;38:14026. doi: 10.3389/ti.2025.14026 (PMC11957988; doi:10.3389/ti.2025.14026)
Supplement: Supplementary file 1 [file DataSheet1.docx]

**Supplementary Material**


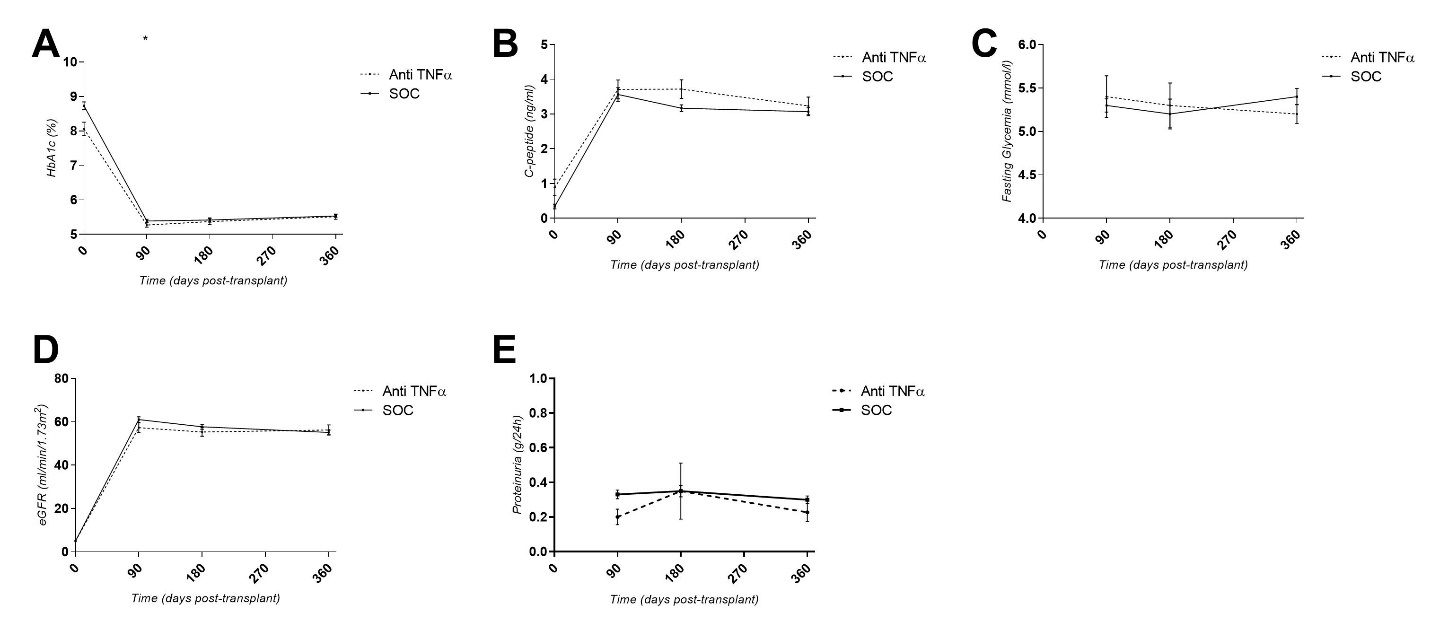


**Figure S1**. **A.** Evolution of HbA1c (%) during the first year post-transplantation according to the administration of anti-TNFα. **B.** Evolution of fasting C-peptide (ng/ml) during the first year post-transplantation according to the administration of anti-TNFα. **C.** Evolution of fasting glycemia (mmol/L) during the first year post-transplantation according to the administration of anti-TNFα. **D.** Evolution of eGFR (CKD-EPI) during the first year post-transplantation according to the administration of anti-TNFα in the SPK subgroup. **E.** Evolution of proteinuria (g/24h) levels during the first year post-transplantation according to the administration of anti-TNFα in the SPK subgroup.

*
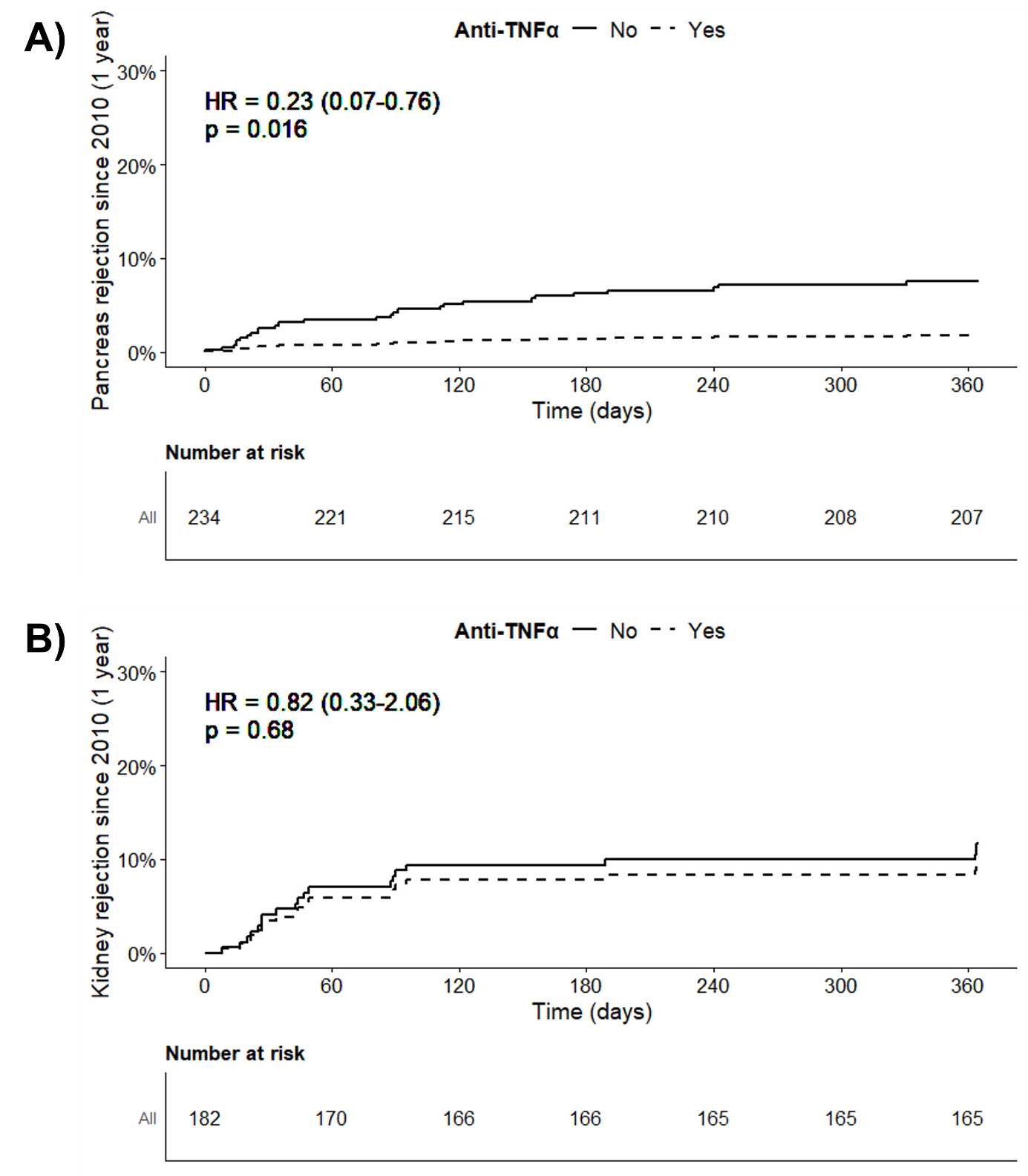
*

**Figure S2**. **A.**Confounder-adjusted death-censored occurrence of pancreas rejection during the first year post-transplant according to the administration of anti-TNFα. **B.** Confounder-adjusted death-censored occurrence of kidney rejection according to the administration of anti-TNFα among SPK recipients during the first year post-transplant

*
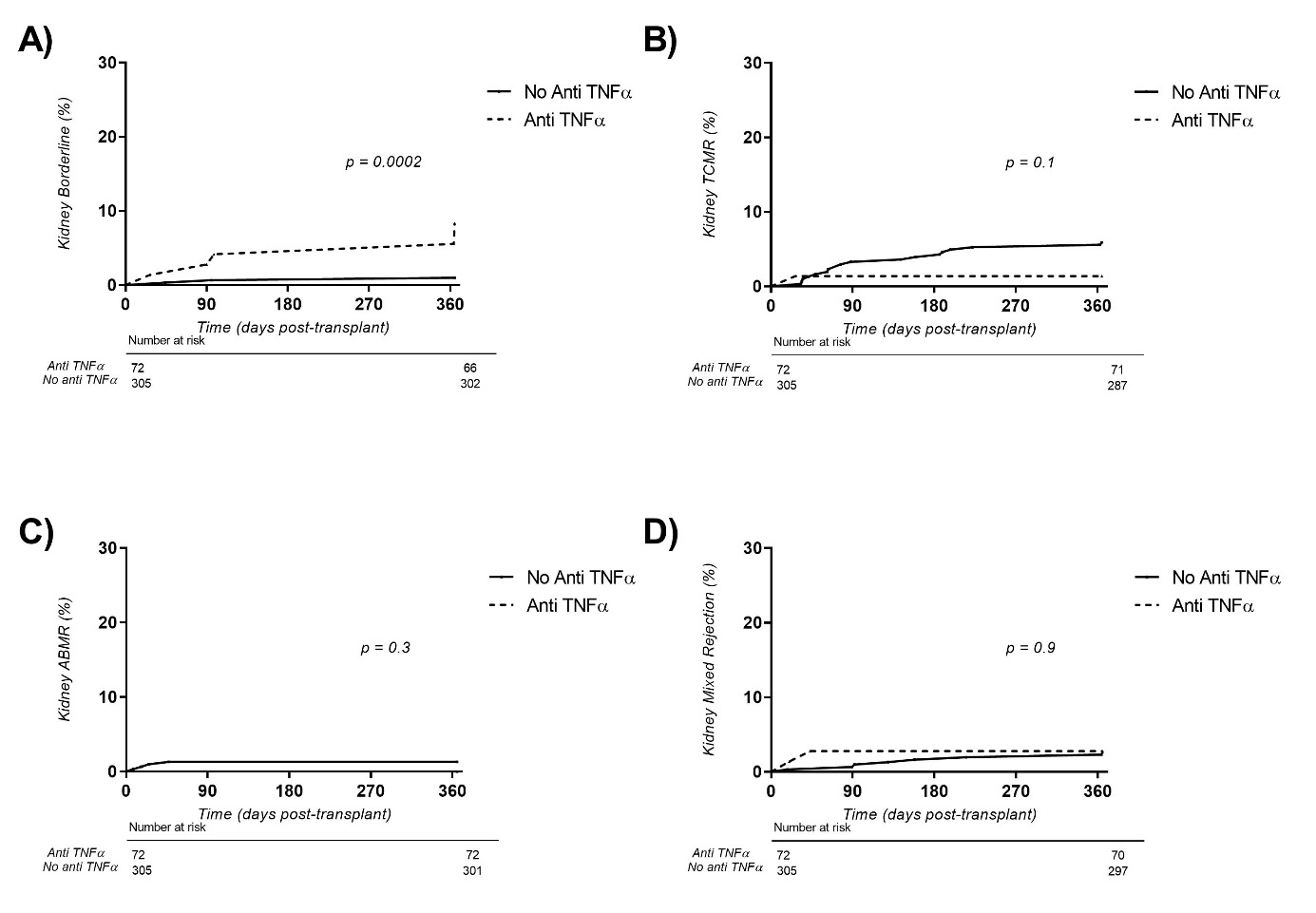
*

**Figure S3** Incidence of Borderline (**A**), TCMR (**B**), ABMR (**C**) and Mixed (**D**) kidney rejection episodes among recipients of SPK during the study period 2000-2022

*Table S1.* Univariate cause-specific Cox model associated with the risk of pancreas graft failure within the first year post-transplantation.

|  | HR | Lower CI95% | Upper CI95% | p |
| --- | --- | --- | --- | --- |
| **Anti-TNFα** | 1.12 | 0.62 | 2.01 | 0.704 |
| **Type of graft : PAK (vs SPK)** | 1.53 | 0.75 | 3.11 | 0.238 |
| **Type of graft : PTA (vs SPK)** | 1.32 | 0.67 | 2.59 | 0.424 |
| **Rank of transplant > 1** | 1.49 | 0.74 | 3 | 0.263 |
| **Recipient BMI** | 1.08 | 1.02 | 1.15 | 0.011 |
| **Positive DSA at transplantation** | 1.46 | 0.63 | 3.39 | 0.378 |
| **Pancreas Cold Ischemia Time** | 1 | 1 | 1 | 0.348 |
| **Donor Age** | 1.01 | 0.99 | 1.03 | 0.3 |
| **Donor BMI** | 1.1 | 1.01 | 1.19 | 0.021 |
| **Vascular cause of death** | 1.03 | 0.64 | 1.64 | 0.907 |
| **Donor history of hypertension** | 1.99 | 0.96 | 4.16 | 0.066 |
| **History of donor cardiac arrest** | 0.66 | 0.34 | 1.29 | 0.222 |
| **Use of vasopressive drugs** | 1.01 | 0.5 | 2.04 | 0.987 |

*Table S2.* Univariate cause-specific Cox model associated with the risk of kidney graft failure within the first year post-transplantation

|  | HR | Lower CI95% | Upper CI95% | p |
| --- | --- | --- | --- | --- |
| **Anti-TNFα** | 1.06 | 0.12 | 9.51 | 0.956 |
| **Recipient Age** | 1.11 | 0.99 | 1.25 | 0.062 |
| **Recipient BMI** | 1.25 | 1.01 | 1.54 | 0.04 |
| **Pretransplant dialysis** | 0.63 | 0.25 | 1.59 | 0.331 |
| **Kidney Cold ischemia time** | 1 | 1 | 1 | 0.866 |
| **Duration of diabetes pre-transplant** | 1.03 | 0.92 | 1.15 | 0.649 |
| **Donor Age** | 1.01 | 0.92 | 1.09 | 0.906 |
| **Donor BMI** | 1.44 | 1.07 | 1.95 | 0.016 |
| **Vascular cause of death** | 0.91 | 0.15 | 5.46 | 0.919 |
| **Donor history of hypertension** | 3.05 | 0.34 | 27.3 | 0.318 |
| **History of donor cardiac arrest** | 1.03 | 0.12 | 9.22 | 0.978 |
| **Donor clearance (CKD-EPI)** | 1.02 | 0.98 | 1.06 | 0.369 |
| **HLA incompatibilities** | 0.79 | 0.37 | 1.7 | 0.544 |
| **Positive DSA at transplantation** | 4.41 | 0.46 | 42.37 | 0.199 |
| **Depleting induction** | 0.17 | 0.02 | 1.54 | 0.116 |

*Table S3*. Multivariate cause-specific Cox model associated with the risk of kidney graft failure within the first year post-transplantation. (5 events were observed during follow-up, 0 observations were excluded because of missing data).

|  | HR | Low HR | High HR | p |
| --- | --- | --- | --- | --- |
| **Anti-TNFα** | 1.06 | 0.119 | 9.511 | 0.9564 |

*Table S4*. Univariate cause-specific Cox model associated with the risk of pancreas graft rejection within the first year post-transplantation (period study 2000-2022).

|  | HR | Lower CI95% | Upper CI95% | p |
| --- | --- | --- | --- | --- |
| **Anti-TNFα** | 0.31 | 0.1 | 1.02 | 0.053 |
| **Type of graft : PAK (vs SPK)** | 3.09 | 1.39 | 6.85 | 0.005 |
| **Type of graft : PTA (vs SPK)** | 3.21 | 1.58 | 6.52 | 0.001 |
| **Recipient Age** | 1 | 0.96 | 1.04 | 0.991 |
| **Recipient Sex** | 1.1 | 0.61 | 2 | 0.749 |
| **Recipient BMI** | 1.06 | 0.98 | 1.14 | 0.155 |
| **Pancreas Cold Ischemia Time** | 1 | 1 | 1 | 0.584 |
| **Donor Age** | 1.02 | 0.99 | 1.05 | 0.226 |
| **Donor Sex** | 1.19 | 0.65 | 2.16 | 0.58 |
| **Donor clearance (CKD-EPI)** | 1 | 0.99 | 1.02 | 0.498 |
| **HLA incompatibilities** | 1.22 | 0.91 | 1.64 | 0.179 |
| **Cyclosporine from D0** | 0.77 | 0.28 | 2.16 | 0.624 |
| **Oral steroid therapy from D0** | 2.21 | 0.87 | 5.6 | 0.096 |
| **Other immunosuppressive treatment** | 5.49 | 1.7 | 17.74 | 0.004 |
| **Anti HLA class I at transplantation** | 1.52 | 0.75 | 3.08 | 0.247 |
| **Anti HLA class II at transplantation** | 0.99 | 0.39 | 2.51 | 0.976 |
| **Positive DSA at transplantation** | 1.09 | 0.34 | 3.54 | 0.885 |
| **Retransplantation** | 1.62 | 0.69 | 3.84 | 0.269 |
| **Depleting induction** | 0.84 | 0.2 | 3.49 | 0.815 |

*Table S5*. Descriptive table of subcohort 2010-2022 depending on the administration of Anti-TNFα in the early post-operative time (p-values are obtained using Chi-square test or Fisher exact test for categorical variables and using Student t-test or Mann-Whitney U for continuous variables).

|  | **Whole cohort (n=236)** | | | **Anti-TNFα (n=87)** | | | **Standard of care (n=149)** | | | **p-value** |
| --- | --- | --- | --- | --- | --- | --- | --- | --- | --- | --- |
|  | **NA** | **n** | **%** | **NA** | **n** | **%** | **NA** | **n** | **%** |  |
| **Type of graft** | 0 |  |  | 0 |  |  | 0 |  |  |  |
| *SPK* |  | 182 | 77.1 |  | 72 | 82.7 |  | 110 | 73.8 | 0.1481 |
| *PAK* |  | 22 | 9.3 |  | 4 | 4.6 |  | 18 | 12.1 | 0.0651 |
| *PTA* |  | 32 | 13.6 |  | 11 | 12.6 |  | 21 | 14.1 | 0.8451 |
| **Male recipient** | 0 | 133 | 56.3 | 0 | 46 | 52.9 | 0 | 87 | 58.4 | 0.4181 |
| **Retransplantation** | 0 | 29 | 12.3 | 0 | 8 | 9.2 | 0 | 21 | 14.1 | 0.3096 |
| **Pancreas conservation fluid** | 13 |  |  | 3 |  |  | 10 |  |  |  |
| *Celsior* |  | 65 | 29.2 |  | 8 | 9.5 |  | 57 | 41.0 | <0.0001 |
| *IGL* |  | 89 | 39.9 |  | 53 | 63.1 |  | 36 | 25.9 | <0.0001 |
| *Other* |  | 69 | 30.9 |  | 23 | 27.4 |  | 46 | 33.1 | 0.4560 |
| **Male donor** | 0 | 157 | 66.5 | 0 | 56 | 64.4 | 0 | 101 | 67.8 | 0.2350 |
| **Vascular cause of donor death** | 0 | 92 | 38.9 | 0 | 34 | 39.1 | 0 | 58 | 38.9 | >0.9999 |
| **Donor hypertension history** | 0 | 16 | 7.2 | 9 | 5 | 6.4 | 5 | 11 | 7.3 | 0.7572 |
| **History of donor cardiac arrest sampling** | 0 | 61 | 25.1 | 1 | 25 | 29.1 | 1 | 36 | 24.3 | 0.4431 |
| **Use of vasopressive drug** | 0 | 203 | 89.4 | 8 | 74 | 93.7 | 1 | 129 | 87.2 | 0.1741 |
| **Depleting induction** | 0 | 218 | 87.5 | 0 | 87 | 100 | 0 | 131 | 87.9 | 0.0002 |
| **Maintenance therapy from Day 0** | 0 |  |  | 0 |  |  |  |  |  |  |
| *Cyclosporine* |  | 2 | 0.8 |  | 0 | 0 | 0 | 2 | 1.3 | 0.5325 |
| *Tacrolimus* |  | 234 | 99.1 |  | 87 | 100 | 0 | 147 | 98.6 | 0.5325 |
| *Antiproliferative drugs* |  | 235 | 99.6 |  | 87 | 100 | 0 | 148 | 99.3 | >0.9999 |
| *mTOR inhibitors* |  | 0 | 0 |  | 0 | 0 | 0 | 0 | 0 | >0.9999 |
| *Oral steroids* |  | 231 | 97.9 |  | 87 | 100 | 0 | 144 | 96.6 | 0.2963 |
| **Positive DSA at transplantation** | 0 | 25 | 10.6 | 0 | 10 | 11.5 | 0 | 15 | 10.4 | 0.6587 |
|  |  |  |  |  |  |  |  |  |  |  |
| **Recipient age (years)** | 0 | 40.6 | 7.9 | 0 | 39.6 | 7.3 | 0 | 41.3 | 8.3 | 0.1104 |
| **Recipient BMI (kg/m²)** | 0 | 23.7 | 3.7 | 0 | 23.9 | 3.8 | 0 | 23.6 | 3.6 | 0.3313 |
| **Duration of diabetes (years)** | 8 | 26.4 | 8.7 | 8 | 24.6 | 8.8 | 0 | 27.4 | 8.5 | 0.0276 |
| **Pancreas cold ischemia time(min)** | 0 | 608 | 140 | 0 | 563 | 136 | 1 | 635 | 136 | <0.0001 |
| **Kidney cold ischemia time (min)** | 0 | 753 | 155 | 0 | 688 | 133 | 0 | 794 | 154 | <0.0001 |
| **Duration in ICU at post-op (days)** | 6 | 1.7 | 1.7 | 6 | 1.4 | 0.9 | 0 | 1.9 | 1.9 | 0.0194 |
| **Donor age (years)** | 0 | 32.9 | 10.9 | 0 | 33.1 | 11.2 | 0 | 32.7 | 10.8 | 0.7978 |
| **Donor BMI (kg/m²)** | 0 | 23.1 | 3.0 | 0 | 22.8 | 2.9 | 0 | 23.2 | 3.1 | 0.4103 |
| **Donor creatininemia (µmol/L)** | 0 | 77 | 33 | 0 | 80 | 40 | 0 | 76 | 28 | 0.8970 |
| BMI, body mass index; eGFR, estimated glomerular filtration rate; ICU, intensive care unit; NA: not available (missing); PAK, pancreas after kidney; PTA, pancreas transplant alone; SD, standard deviation; SPK, simultaneous pancreas-kidney. | | | | | | | | | | |

*Table S6*. Univariate cause-specific Cox model associated with the risk of pancreas graft rejection within the first year post-transplantation (period study 2010-2022).

|  | HR | Lower CI95% | Upper CI95% | p |
| --- | --- | --- | --- | --- |
| **Anti-TNFα** | 0.2 | 0.06 | 0.66 | 0.008 |
| **Type of graft : PAK (vs SPK)** | 4.36 | 1.65 | 11.46 | 0.003 |
| **Type of graft : PTA (vs SPK)** | 3.96 | 1.64 | 9.56 | 0.002 |
| **Recipient Age** | 1 | 0.96 | 1.05 | 0.93 |
| **Recipient Sex** | 1.2 | 0.57 | 2.56 | 0.631 |
| **Recipient BMI** | 1.05 | 0.95 | 1.15 | 0.335 |
| **Donor Age** | 1.04 | 1 | 1.07 | 0.043 |
| **Donor Sex** | 2.28 | 1.07 | 4.86 | 0.032 |
| **Donor BMI** | 1.12 | 0.99 | 1.27 | 0.077 |
| **HLA incompatibilities** | 1.2 | 0.8 | 1.78 | 0.376 |
| **Cyclosporine from D0** | 7.37 | 2.54 | 21.35 | 0.001 |
| **Oral steroid therapy from D0** | 1.14 | 0.15 | 8.41 | 0.897 |
| **Other immunosuppressive treatment** | 4.34 | 1.3 | 14.41 | 0.017 |
| **Anti HLA class I at transplantation** | 1.72 | 0.77 | 3.85 | 0.19 |
| **Anti HLA class II at transplantation** | 0.86 | 0.32 | 2.3 | 0.768 |
| **Positive DSA at transplantation** | 0.97 | 0.29 | 3.23 | 0.954 |
| **Retransplantation** | 2.19 | 0.88 | 5.42 | 0.091 |
| **Depleting induction** | 1.02 | 0.24 | 4.29 | 0.983 |

*Table S7*. Multivariate cause-specific Cox model associated with the risk of pancreas graft rejection within the first year post-transplantation (period study 2010-2022, 27 events were observed during follow-up, 0 observations were excluded because of missing data).

|  | HR | Low HR | High HR | p |
| --- | --- | --- | --- | --- |
| **Anti-TNFα** | 0.23 | 0.067 | 0.758 | 0.0161 |
| **Type of graft : SPK** | 0.29 | 0.132 | 0.621 | 0.0015 |
| **Depletant induction therapy** | 0.96 | 0.219 | 4.207 | 0.9569 |
| **Donor Sex** | 2.31 | 1.082 | 4.950 | 0.0305 |

*Table S8*. Univariate cause-specific Cox model associated with the risk of kidney rejection within the first year post-transplantation in the SPK subgroup.

|  | HR | Lower CI95% | Upper CI95% | p |
| --- | --- | --- | --- | --- |
| **Anti-TNFα** | 1.01 | 0.44 | 2.29 | 0.99 |
| **Recipient Age** | 1 | 0.96 | 1.05 | 0.908 |
| **Recipient Sex** | 0.58 | 0.28 | 1.2 | 0.144 |
| **Recipient BMI** | 1.02 | 0.93 | 1.12 | 0.616 |
| **Pretransplant dialysis** | 1.09 | 0.77 | 1.54 | 0.627 |
| **Kidney Cold ischemia time** | 1 | 1 | 1 | 0.551 |
| **Duration of diabetes pre-transplant** | 0.98 | 0.94 | 1.03 | 0.404 |
| **Donor Age** | 1.01 | 0.98 | 1.05 | 0.355 |
| **Donor BMI** | 0.82 | 0.41 | 1.63 | 0.565 |
| **Duration of diabetes pre-transplant** | 1.08 | 0.96 | 1.21 | 0.197 |
| **Vascular cause of death** | 1.18 | 0.62 | 2.25 | 0.616 |
| **Donor history of hypertension** | 2.5 | 1.04 | 6 | 0.04 |
| **History of donor cardiac arrest** | 0.98 | 0.43 | 2.22 | 0.955 |
| **Donor clearance (CKD-EPI)** | 1 | 0.98 | 1.01 | 0.511 |
| **HLA incompatibilities** | 1.2 | 0.88 | 1.65 | 0.244 |
| **Cyclosporine from D0** | 0.82 | 0.29 | 2.33 | 0.714 |
| **Oral steroid therapy from D0** | 0.97 | 0.42 | 2.21 | 0.938 |
| **Positive DSA at transplantation** | 1.41 | 0.43 | 4.61 | 0.572 |
| **Depleting induction** | 0.44 | 0.13 | 1.42 | 0.168 |

*Table S9*. Multivariate cause-specific Cox model associated with the risk of kidney rejection within the first year post-transplantation in the SPK subgroup. (37 events were observed during follow-up, 0 observations were excluded because of missing data).

|  | HR | Low HR | High HR | p |
| --- | --- | --- | --- | --- |
| **Anti-TNFα** | 1.01 | 0.442 | 2.289 | 0.9895 |

*Table S10.* Univariate cause-specific Cox model associated with the risk of severe bacterial infection within the first year post-transplantation

|  | HR | Lower CI95% | Upper CI95% | p |
| --- | --- | --- | --- | --- |
| **Anti-TNFα** | 0.81 | 0.6 | 1.1 | 0.17 |
| **Type of graft : PAK (vs SPK)** | 0.86 | 0.56 | 1.31 | 0.477 |
| **Type of graft : PTA (vs SPK)** | 0.76 | 0.52 | 1.12 | 0.161 |
| **Recipient Age** | 1 | 0.99 | 1.02 | 0.959 |
| **Recipient Sex** | 1.24 | 0.98 | 1.56 | 0.072 |
| **Recipient BMI** | 1 | 0.96 | 1.03 | 0.793 |
| **Pancreas Cold Ischemia Time** | 1 | 1 | 1 | 0.526 |
| **Donor Age** | 1 | 0.99 | 1.01 | 0.858 |
| **Donor Sex** | 0.82 | 0.64 | 1.04 | 0.1 |
| **Donor BMI** | 1.03 | 0.99 | 1.07 | 0.212 |
| **Vascular cause of death** | 0.86 | 0.68 | 1.08 | 0.2 |
| **Cyclosporine from D0** | 0.67 | 0.45 | 1 | 0.048 |
| **Oral steroid therapy from D0** | 1.26 | 0.94 | 1.68 | 0.122 |
| **Anti HLA class I at transplantation** | 1.16 | 0.85 | 1.59 | 0.346 |
| **Anti HLA class II at transplantation** | 1.36 | 0.97 | 1.92 | 0.077 |
| **Positive DSA at transplantation** | 1.67 | 1.08 | 2.56 | 0.02 |
| **Retransplantation** | 0.94 | 0.62 | 1.41 | 0.753 |
| **Depleting induction** | 0.96 | 0.54 | 1.71 | 0.881 |

*Table S11*. Multivariate cause-specific Cox model associated with the risk of severe bacterial infection within the first year post-transplantation. (291 events were observed during follow-up, 2 observations were excluded because of missing data).

|  | HR | Low HR | High HR | p |
| --- | --- | --- | --- | --- |
| **Anti-TNFα** | 0.74 | 0.539 | 1.005 | 0.0536 |
| **Recipient Sex** | 1.29 | 1.018 | 1.625 | 0.0351 |
| **Donor Sex** | 0.79 | 0.619 | 1.006 | 0.0562 |
| **Cyclosporine from D0** | 0.61 | 0.407 | 0.918 | 0.0178 |

*Table S12*. Univariate cause-specific Cox model associated with the risk of fungal infection within the first year post-transplantation

|  | HR | Lower CI95% | Upper CI95% | p |
| --- | --- | --- | --- | --- |
| **Anti-TNFα** | 1.27 | 0.61 | 2.67 | 0.522 |
| **Type of graft : PAK (vs SPK)** | 1.11 | 0.39 | 3.13 | 0.847 |
| **Type of graft : PTA (vs SPK)** | 1.04 | 0.41 | 2.67 | 0.933 |
| **Recipient Age** | 1.03 | 0.99 | 1.07 | 0.098 |
| **Recipient Sex** | 0.93 | 0.5 | 1.75 | 0.833 |
| **Recipient BMI** | 1.04 | 0.96 | 1.13 | 0.323 |
| **Pancreas Cold Ischemia Time** | 1 | 1 | 1 | 0.007 |
| **Duration of diabetes** | 1.06 | 1.02 | 1.1 | 0.006 |
| **Donor Age** | 0.99 | 0.96 | 1.02 | 0.554 |
| **Donor Sex** | 1.08 | 0.58 | 2.03 | 0.803 |
| **Donor BMI** | 0.98 | 0.88 | 1.1 | 0.77 |
| **Vascular cause of death** | 0.96 | 0.51 | 1.78 | 0.891 |
| **Cyclosporine from D0** | 0.19 | 0.03 | 1.36 | 0.097 |
| **Oral steroid therapy from D0** | 5.57 | 1.35 | 23.09 | 0.018 |
| **Anti HLA class I at transplantation** | 2.48 | 1.25 | 4.94 | 0.01 |
| **Anti HLA class II at transplantation** | 1.36 | 0.57 | 3.27 | 0.486 |
| **Positive DSA at transplantation** | 1.17 | 0.36 | 3.81 | 0.796 |
| **Retransplantation** | 1.07 | 0.38 | 3.01 | 0.894 |
| **Depleting induction** | 0.5 | 0.15 | 1.63 | 0.25 |

*Table S13*. Multivariate cause-specific Cox model associated with the risk of fungal infection within the first year post-transplantation. (41 events were observed during follow-up, 13 observations were excluded because of missing data).

|  | HR | Low HR | High HR | p |
| --- | --- | --- | --- | --- |
| **Anti-TNFα** | 0.82 | 0.373 | 1.799 | 0.6187 |
| **Pancreas Cold Ischemia Time** | 0.99 | 0.995 | 0.999 | 0.0072 |

*Table S14.* Univariate cause-specific Cox model associated with the risk of CMV viremia within the first year post-transplantation

|  | HR | Lower CI95% | Upper CI95% | p |
| --- | --- | --- | --- | --- |
| **Anti-TNFα** | 1.29 | 0.68 | 2.44 | 0.44 |
| **Type of graft : PAK (vs SPK)** | 0.82 | 0.29 | 2.27 | 0.696 |
| **Type of graft : PTA (vs SPK)** | 1.46 | 0.71 | 2.99 | 0.306 |
| **Recipient Age** | 0.98 | 0.95 | 1.02 | 0.32 |
| **Recipient Sex** | 1.06 | 0.62 | 1.81 | 0.836 |
| **Recipient BMI** | 0.97 | 0.89 | 1.04 | 0.388 |
| **Pancreas Cold Ischemia Time** | 1 | 1 | 1 | 0.001 |
| **Duration of diabetes** | 1 | 0.97 | 1.04 | 0.865 |
| **Inferior vena cava anastomosis** | 6.11 | 2.61 | 14.27 | 0.001 |
| **Donor Age** | 1.01 | 0.98 | 1.03 | 0.479 |
| **Donor Sex** | 1.44 | 0.85 | 2.44 | 0.181 |
| **Donor BMI** | 1.05 | 0.95 | 1.15 | 0.333 |
| **Vascular cause of death** | 1.44 | 0.85 | 2.44 | 0.179 |
| **Cyclosporine from D0** | 1.36 | 0.64 | 2.88 | 0.419 |
| **Oral steroid therapy from D0** | 2.85 | 1.14 | 7.16 | 0.025 |
| **Anti HLA class I at transplantation** | 1.17 | 0.59 | 2.32 | 0.662 |
| **Anti HLA class II at transplantation** | 2.87 | 1.55 | 5.29 | 0.001 |
| **Positive DSA at transplantation** | 1.83 | 0.78 | 4.3 | 0.163 |
| **Retransplantation** | 1.44 | 0.65 | 3.18 | 0.368 |
| **Depleting induction** | 0.69 | 0.21 | 2.2 | 0.527 |

*Table S15.* Multivariate cause-specific Cox model associated with the risk of CMV viremia within the first year post-transplantation (54 events were observed during follow-up, 50 observations were excluded because of missing data).

|  | HR | Low HR | High HR | p |
| --- | --- | --- | --- | --- |
| **Anti-TNFα** | 0.89 | 0.454 | 1.741 | 0.7310 |
| **Inferior vena cava anastomosis** | 6.27 | 2.65 | 14.838 | <0.0001 |

*Table S16*. Univariate cause-specific Cox model associated with the risk of BKV viremia within the first year post-transplantation

|  | HR | Lower CI95% | Upper CI95% | p |
| --- | --- | --- | --- | --- |
| **Anti-TNFα** | 1.43 | 0.79 | 2.6 | 0.242 |
| **Type of graft : PAK (vs SPK)** | 0.98 | 0.39 | 2.47 | 0.964 |
| **Type of graft : PTA** | 1.56 | 0.79 | 3.09 | 0.204 |
| **Recipient Age** | 1 | 0.97 | 1.03 | 0.866 |
| **Recipient Sex** | 1.03 | 0.61 | 1.72 | 0.92 |
| **Recipient BMI** | 1.01 | 0.94 | 1.09 | 0.7 |
| **Pancreas Cold Ischemia Time** | 1 | 1 | 1 | 0.001 |
| **Donor Age** | 1.01 | 0.98 | 1.03 | 0.645 |
| **Donor Sex** | 1.04 | 0.62 | 1.75 | 0.888 |
| **Donor BMI** | 1.02 | 0.93 | 1.11 | 0.716 |
| **Vascular cause of death** | 0.81 | 0.48 | 1.36 | 0.43 |
| **Cyclosporine from D0** | 0.58 | 0.21 | 1.59 | 0.289 |
| **Oral steroid therapy from D0** | 5.39 | 1.69 | 17.24 | 0.004 |
| **Anti HLA class I at transplantation** | 1.23 | 0.64 | 2.38 | 0.537 |
| **Anti HLA class II at transplantation** | 1.42 | 0.7 | 2.9 | 0.333 |
| **Positive DSA at transplantation** | 0.52 | 0.13 | 2.13 | 0.36 |
| **Retransplantation** | 1.35 | 0.62 | 2.98 | 0.452 |
| **Depleting induction** | 0.33 | 0.14 | 0.78 | 0.011 |

*Table S17*. Multivariate cause-specific Cox model associated with the risk of BKV viremia within the first year post-transplantation. (58 events were observed during follow-up, 2 observations were excluded because of missing data).

|  | HR | Low HR | High HR | p |
| --- | --- | --- | --- | --- |
| **Anti-TNFα** | 0.97 | 0.513 | 1.844 | 0.9329 |
| **Oral steroid therapy from D0** | 5.43 | 1.684 | 17.475 | 0.0046 |
